# A Quasi-Physiological Microfluidic Blood-Brain Barrier Model for Brain Permeability Studies

**DOI:** 10.3390/pharmaceutics13091474

**Published:** 2021-09-15

**Authors:** Behnam Noorani, Aditya Bhalerao, Snehal Raut, Ehsan Nozohouri, Ulrich Bickel, Luca Cucullo

**Affiliations:** 1 Department of Pharmaceutical Sciences, Jerry H. Hodge School of Pharmacy, Texas Tech University Health Sciences Center, Amarillo, TX 79106, USA; behnam.noorani@ttuhsc.edu (B.N.); ehnozoho@ttuhsc.edu (E.N.); 2 Center for Blood-Brain Barrier Research, School of Pharmacy, Texas Tech University Health Sciences Center, Amarillo, TX 79106, USA; 3 Department of Biological and Biomedical Sciences, Oakland University, Rochester, MI 48309, USA; abhalerao@oakland.edu; 4 Department of Foundational Medical Studies, Oakland University William Beaumont School of Medicine, Rochester, MI 48309, USA; sraut@oakland.edu

**Keywords:** endothelium, alternative, drug discovery, pericytes, stem cells, co-culture, shear stress, NVU, in vitro

## Abstract

Microfluidics-based organ-on-a-chip technology allows for developing a new class of in-vitro blood-brain barrier (BBB) models that recapitulate many hemodynamic and architectural features of the brain microvasculature not attainable with conventional two-dimensional platforms. Herein, we describe and validate a novel microfluidic BBB model that closely mimics the one in situ. Induced pluripotent stem cell (iPSC)-derived brain microvascular endothelial cells (BMECs) were juxtaposed with primary human pericytes and astrocytes in a co-culture to enable BBB-specific characteristics, such as low paracellular permeability, efflux activity, and osmotic responses. The permeability coefficients of [^13^C_12_] sucrose and [^13^C_6_] mannitol were assessed using a highly sensitive LC-MS/MS procedure. The resulting BBB displayed continuous tight-junction patterns, low permeability to mannitol and sucrose, and quasi-physiological responses to hyperosmolar opening and p-glycoprotein inhibitor treatment, as demonstrated by decreased BBB integrity and increased permeability of rhodamine 123, respectively. Astrocytes and pericytes on the abluminal side of the vascular channel provided the environmental cues necessary to form a tight barrier and extend the model’s long-term viability for time-course studies. In conclusion, our novel multi-culture microfluidic platform showcased the ability to replicate a quasi-physiological brain microvascular, thus enabling the development of a highly predictive and translationally relevant BBB model.

## 1. Introduction

Central nervous system (CNS) disorders have limited treatment options, although they are significant causes of morbidity and mortality worldwide [1]. A considerable obstacle in developing CNS drug therapeutics is the BBB, restricting nearly 98% of small molecules and virtually all macromolecules from entering the brain [2]. The BBB, a unique physiological feature in vertebrates, is responsible for maintaining brain homeostasis [3,4]. Its components are BMECs, astrocytes, pericytes, neurons, and the extracellular matrix (ECM) [5]. The structural and functional unit derived from this grouping of cells and the surrounding matrix is known as the neurovascular unit (NVU) [4]. The BBB is highly selective in controlling the passage of substances between the blood and the CNS. The BMECs that line the vascular bed of the capillaries in the brain possess specialized tight junctions (TJs) that severely limit the paracellular diffusion of polar molecules and efflux pumps that allow for tight control of the transcellular passage of a large variety of lipophilic substances. These gating features allow essential nutrients and molecules to pass through, while preventing harmful substances and most drugs from entering the brain. However, substances can cross the BBB by passive diffusion, adsorptive transcytosis, and receptor- or carrier-mediated transport [6].

BBB impairment is implicated in the pathogenesis and progression of various neurological diseases [7,8,9,10]. Understanding the structural and functional aspects of the BBB assumes utmost clinical importance due to the inability of most drugs to cross the barrier. Traditionally, BBB-related studies have been performed using in vitro models by culturing different cell types individually or together across semi-permeable membranes. However, conventional models cannot recapitulate physiologically relevant conditions, such as blood flow, cell–cell interactions, and the complex three-dimensional (3D) microenvironment of the human BBB [11]. In vivo techniques and animal models have provided more definitive data regarding the BBB. However, these data may not be clinically relevant or translatable due to the interspecies differences between humans and animals [12,13]. In addition, animal studies are expensive and labor intensive and involve ethical considerations [14]. Therefore, modeling the most accurate human representation of the BBB is highly desirable.

Microfluidics-based organ-on-a-chip technology has led to the recent development of a new class of in vitro models [15,16]. These miniature systems are engineered to recreate an environment similar to the microvessels in the human brain [17]. Organs-on-chips enable dynamic growth, function, and interaction of multiple cell types, while enabling perfusion and shear stress. In addition to basic mechanistic and translational studies, microfluidics can be used for drug permeability assessments as a far more accurate alternative to the classic Transwell set-up. Due to their structural advantages, organs-on-chips are usually better for establishing in-vitro–in-vivo correlations for drug permeability measurements. Recent studies have reported the use of iPSC-derived BMECs (iBMECs) in organ-on-a-chip BBB platforms exhibiting near-physiological barrier features [4,18,19,20,21]. However, most research groups have relied on large-molecular-weight dextrans (3–70 kDa) to measure passive permeability [18,19]. This is problematic because the calculation of permeability coefficients without considering the size of the selected dextran may not effectively capture the integrity of the BBB on a chip. The permeability coefficient of dextran reported by Yuan et al. [22] using in vivo fluorescence imaging analysis has been repeatedly cited as a reference point for correlation studies. However, passive diffusion of dextran fluorescent markers crossing the BBB in vivo is highly unlikely due to its molecular weight. In fact, even most of the small-molecular-weight-drug candidates are unable to pass the BBB. As such, it is essential to use small-molecular-weight markers to validate the barrier function in these advanced BBB models. We have previously demonstrated the use of an LCMS method using sucrose (353 Da) and mannitol (187 Da) as superior alternatives for BBB permeability studies [23,24]. They possess high metabolic stability, do not interact with proteins, and are uncharged, enabling precise paracellular BBB permeability measurement [23,24,25,26,27].

This study validated the barrier function of a BBB-on-a-chip platform using [^13^C_12_] sucrose and [^13^C_6_] mannitol. We co-cultured iBMECs with primary human pericytes and astrocytes on the organ-on-a-chip model to emulate BBB-specific characteristics, such as high barrier function, low permeability, and efflux activity. We also showed that the organ-on-a-chip model could maintain the barrier function for 1 week, whereas traditional iBMECs Transwell models can maintain the barrier function for only 2 days.

## 2. Materials and Methods

### 2.1. Cell Culture

Primary human brain astrocytes (ScienCell # 1800, Carlsbad, CA, USA) and primary human brain pericytes (Cell Systems # ACBRI 498, Kirkland, WA, USA) were grown in the recommended growth medium. Primary cells were used at passages 1–3. The iPSC (IMR90) clone cell line (Wicell # iPS(IMR90)-4, Madison, WI, USA) with passage number (40–55) was used to derive BMECs.

### 2.2. iPSCs Differentiation to iBMECs

iPSCs were differentiated into iBMECs as per existing protocol [28,29]. Briefly, iPS (IMR90)-4 were grown in Essential 8 medium (Thermo Fisher Scientific # A1517001, Waltham, MA, USA) containing 10 μM Y-27632 dihydrochloride (Tocris # 1254). After cells reached 70% confluency, differentiation into iBMECs was initiated using unconditioned medium (Thermo Fisher Scientific # 11330057), 20% knockout serum replacement (Thermo Fisher Scientific # 10828010), 1% non-essential amino acids (Thermo Fisher Scientific # 11140050), 0.5% Glutamax (Thermo Fisher Scientific # 35050079), and 0.1 mM β-mercaptoethanol (Sigma-Aldrich # M6250, St. Louis, MO, USA), which continued for 6 days. Subsequently, the media were changed to EC++ media, i.e., human endothelial SFM (Thermo Fisher Scientific # 11111044) with 1% platelet-poor plasma-derived serum, bovine (Alfa Aesar # 15406419, Haverhill, MA, USA), 20 ng/mL bFGF, and 10 μM retinoic acid (Sigma-Aldrich # R2625). After 2 days, the medium was replaced with EC (SFM without bFGF and retinoic acid). The cells were harvested using Accutase (Corning # 25-058-CI, Corning, NY, USA) for seeding on a BBB on a chip.

### 2.3. BBB on a Chip

We used a commercially available brain on a chip from Emulate, Inc. (Boston, MA, USA). The chip consists of a polydimethylsiloxane (PDMS)-based microfluidic device containing two overlapping microchannels (1 × 1 mm and 1 × 0.2 mm, brain and blood channel, respectively) separated by a permeable PDMS membrane [18,30,31]. The inner surface of the channels was coated with collagen (Sigma # C5533, Kawasaki, Kanagawa) and fibronectin (Sigma # F1141) solution (4:1) in distilled water and incubated overnight at 37 °C. To mimic the physiological microenvironment in the brain, primary astrocytes and pericytes were co-cultured at a 3:1 ratio in the apical channel (1 × 10^6^ mL^−1^ and 3.5 × 10^5^ cells mL^−1^ for astrocytes and pericytes, respectively). The cells were allowed to adhere to the apical channel in the incubator for 4 h. 1.5 × 10^7^ cells mL^−1^ of iBMECs were then seeded on the basal channel, and the chip was turned upside down to allow them to adhere to the permeable PDMS membrane. The device was then turned back to the upright position to allow the remaining iBMECs to adhere to the sides and bottom of the channel, thereby forming a capillary lumen. The flow was introduced the next day with the help of a peristaltic pump (Ismatec, Opfikon, Switzerland) at a rate of 120 μL/h. Experiments were then performed after the cells were adapted to the flow conditions.

### 2.4. Permeability Measurement of Sucrose and Mannitol

Barrier integrity of a BBB microfluidic chip was obtained by measuring the permeability coefficients of stable isotopes of [^13^C_12_] sucrose and [^13^C_6_] mannitol. For this purpose, 100 μg/mL of [^13^C_6_] mannitol and [^13^C_12_] sucrose (Omicron Biochemicals, South Bend, IN, USA) was added simultaneously to the vascular (lumen) channel. Samples were collected over specific time intervals from the apical channel (ablumen) and measured using an established UPLC-MS/MS method [23]. Briefly, the collected samples were diluted in LC-MS/MS-grade water in the standard curve range (2–1000 ng/mL). Samples were then subjected to a protein crashing step by diluting at a ratio of 1:9 in acetonitrile:water (80:20) (Fisher Scientific, USA) containing 20 ng/mL of [^2^H_8_] mannitol and [^2^H_2_] sucrose (Omicron Biochemicals, South Bend, IN, USA) as an internal standard followed by centrifugation at 12,000 rpm for 10 min. The supernatant was transferred into autosampler inserts and then injected into the LC-MS/MS. An Acquity BEH amide column (2.1 mm × 50 mm, 1.7 µm; Waters, Milford, MA, USA) was used for chromatographic separation using acetonitrile:water:ammonium hydroxide (73:27:0.1, *v*/*v*) as the mobile phase, at a flow rate of 0.2 mL/min. Electrospray ionization in negative mode was used as the ionization source, and data acquisition and quantification were made using Analyst software.

The concentration measured through LCMS were used to calculate the permeability coefficient (cm/s) as follows based on a previous study [18]:

(1) $$P=\frac{Cr\times Va}{Cd\times A\times t}$$

Here, *P* is the permeability coefficient (cm/s), *C_r_* is the measured concentration of the marker in the brain effluent at a time (*t*), and *C_d_* is the measured concentration of the marker in the dosing (vascular) channel effluent at a time (*t*). *V_a_* is the volume of the receiving channel at a time (*t*). The membrane area (*A*) is 0.17 cm^2^ in the microfluidic chip, and the flow rate of 120 µL/h was used for all permeability studies.

### 2.5. Immunofluorescence Microscopy

Immunocytochemistry was performed as previously described [18,29]. The channels of the BBB chip were fixed with paraformaldehyde (4% in PBS) for 10 min and then washed. Cells were permeabilized with 0.1% Triton X-100 in PBS and blocked with 10% goat serum in PBS for 30 min. The primary antibodies used to stain the cells are listed in the Supplementary File (Table S1). After overnight incubation, the chips were washed and stained with secondary antibodies conjugated to Alexa Fluor-488 and Alexa Fluor-555. A confocal microscope (A1R, Nikon, NY, USA) was used to obtain the images with a 10× objective.

### 2.6. Dynamic Flow and Shear Stress

The confluent endothelial monolayer in the presence of primary human pericytes and astrocytes was exposed to intravascular medium flow using a peristaltic pump to evaluate the effects of shear stress on barrier function. Microfluidic chips were exposed to different flow rates to simulate different levels of shear stress. Initially, the flow rate was set to 120 µL/h for 1 h and then increased to 1200 and 2400 µL/h to produce higher levels of shear stress. The flow rate was maintained for 48 h. Permeability experiments and immunofluorescence were performed after 48 h of exposure to dynamic flow.

The shear stress was obtained from the following equation [18], where τ is the shear stress (dyn/cm^2^) generated in the vascular channel. Q and μ are the flow rate (μL/min) and the viscosity (Pa.s), respectively.

(2) $$\tau \;=6Q\mu /Wh^{2}$$

The width (*W*) and height (*h*) of the vascular channels were, respectively, 1 mm and 200 μm. The viscosity of the cell culture medium was 3–4 cP after adding 3.5% dextran to the medium.

### 2.7. Efflux Study of the Chip

The P-gp functionality was evaluated using rhodamine 123 (Sigma-Aldrich), a substrate of the P-gp transporter [19,29]. Both channels were pretreated with 5 µM cyclosporine A (CsA, P-gp inhibitor, Sigma-Aldrich) for 1 h. Rhodamine 123 was added to the vascular channel and incubated in the presence or absence of cyclosporine A at a flow rate of 120 µL/h. [^13^C_12_] Sucrose (100 µg/mL) was simultaneously added to monitor the barrier integrity. Effluents were collected from the apical and basal channels and quantified using fluorescence intensity. The fluorescence was measured at 485/530 nm to quantify the rhodamine 123 concentration via a SynergyMX2 ELISA plate reader (BioTek Instruments, Winooski, VT, USA). The BBB permeability of rhodamine 123 and sucrose was measured in the presence or absence of the inhibitor.

### 2.8. BBB Opening Using the Hyperosmolar Solution

A hypertonic solution containing 25% mannitol was introduced into the vascular channel at a 120 μL/h flow rate for 10 min. Mannitol was then replaced with 100 μg/mL of [^13^C_12_] sucrose and [^13^C_6_] mannitol. The permeability coefficients of the markers were obtained at different time points to evaluate the BBB opening and recovery.

### 2.9. Statistical Analyses

Prism 9 (GraphPad Software, La Jolla, CA) was used for the statistical analysis of the data. At least three biological replications were used for all experiments. Student’s unpaired two-tailed *t*-test was used for a comparison of the two groups. Data with more than two groups were analyzed by one-way ANOVA, followed by Tukey’s multiple comparisons test. In all cases, a *p*-value < 0.05 was considered significant. Data were presented as the mean ± SD or individual values.

## 3. Results

### 3.1. Characterization of the Blood-Brain Barrier Microfluid Model

**Figure 1:**
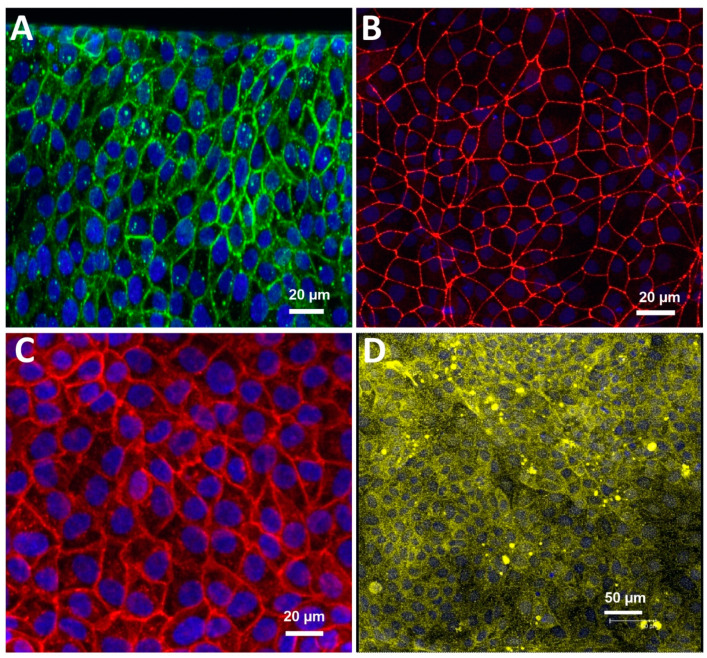
Immunofluorescence images presenting key markers of iBMEC microfluidic chip on day 3. (**A**) Claudin-5, (**B**) ZO-1, (**C**) glucose transporter-1 (GLUT1), and (**D**) P-glycoprotein (P-gp). Nuclei visualized with 4′,6-diamidino-2-phenylindole (DAPI) in blue.

We performed our experiments using the commercially available brain chip (Emulate). It is an in vitro model made of clear PDMS elastomer that artificially recreates a section of the brain vasculature and its surrounding microenvironment. It consists of two superimposed microchannels with a porous membrane in between to allow a cellular interface. We activated and coated the BBB chip as per the manufacturer’s instructions. iBMECs were seeded on the basal channel, and primary astrocytes and pericytes (seeding ratio of 3:1) were added to the apical channel. The iBMECs formed a confluent monolayer of tightly packed cells throughout the basal channel, representing the 3D vascular lumen. BBB-endothelial-specific markers, such as the TJ proteins zona occludens-1 (ZO-1), claudin-5, glucose transporter GLUT-1, and efflux transporter P-gp, were expressed in the channel. Figure 1 shows the iBMEC expression of these key markers inside the BBB on a chip. Figure S1 reveals that the iBMECs formed a continuous band of ZO-1, Glu-1, and Pg-p lining the entire luminal side, thereby forming a blood-vessel-like structure featuring an uninterrupted cellular layer. Note the 3D cross section of the BBB, also shown in Figure S1.

**Figure 2:**
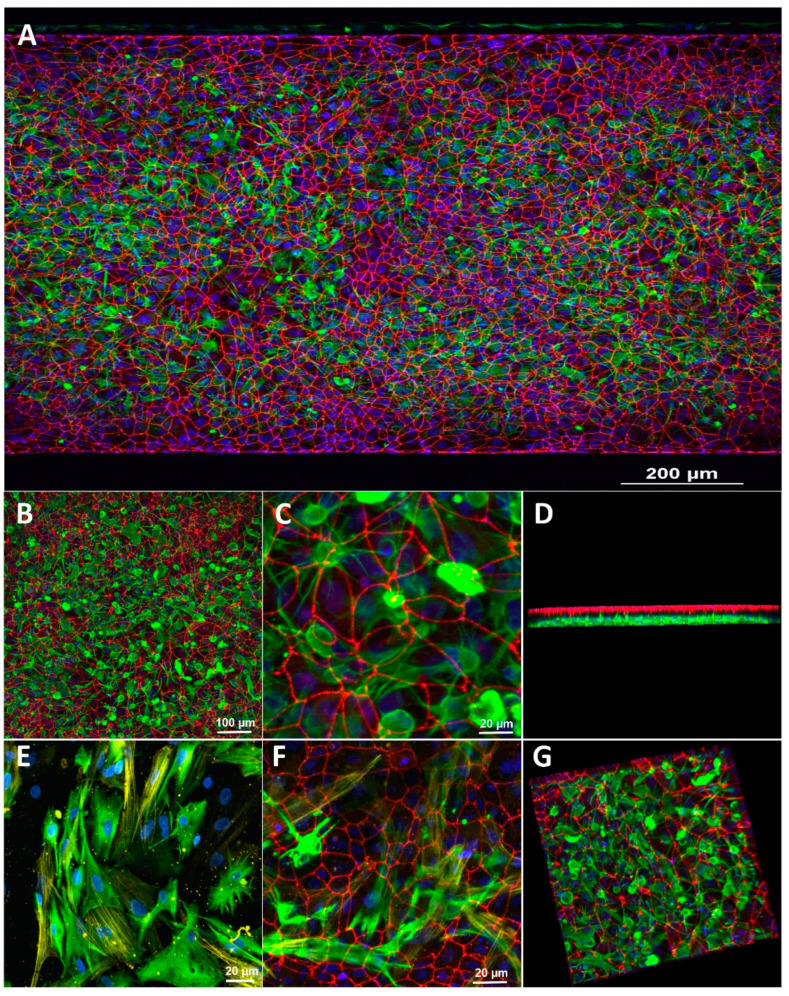
Immunofluorescence images demonstrating the co-culture and triculture of the BBB on a chip. iBMECs cultured on all surfaces of the blood channel, and primary human brain pericytes and astrocytes on the apical brain channel. (**A**–**C**) Different magnifications of iBMECs stained with ZO-1 (red); iBMECs form a tight barrier on the blood side; astrocytes were stained with GFAP (green). (**D**,**G**) 3D structure of the co-culture. (**E**) Co-culture staining of primary human astrocytes (GFAP, green) and primary human pericytes (α-SMA, yellow) in the brain channel. (**F**) Triculture staining of the BBB on a chip (red, iBMECs-ZO-1; green, astrocytes (GFAP); yellow, pericytes (α-SMA)).

Astrocytes and pericytes also grew throughout the apical channel. Glial fibrillary acidic protein (GFAP) and alpha-smooth muscle actin (α-SMA) were used as fluorescent markers for astrocytes and pericytes to distinguish them (Figure 2) visually. The immunocytochemistry imaging of the ZO-1 distribution showed that the iBMECs formed a uniform monolayer covering the entire luminal side of the vascular channel (Figure 2A). Noteworthy is also the distribution of astrocytes and pericytes in the “brain” channel, clearly distinguishable based on the expression of GFAP and α-SMA, respectively. Observation by confocal microscopy confirmed that the iBMECs, astrocytes, and pericytes remained confined to their corresponding channels. Note also that the astrocytic endfeet extended through the permeable membrane to establish contact with the juxtapose endothelial layer in the vascular channel.

### 3.2. Assessment of Paracellular Permeability across a BBB Microfluidic Chip

**Figure 3:**
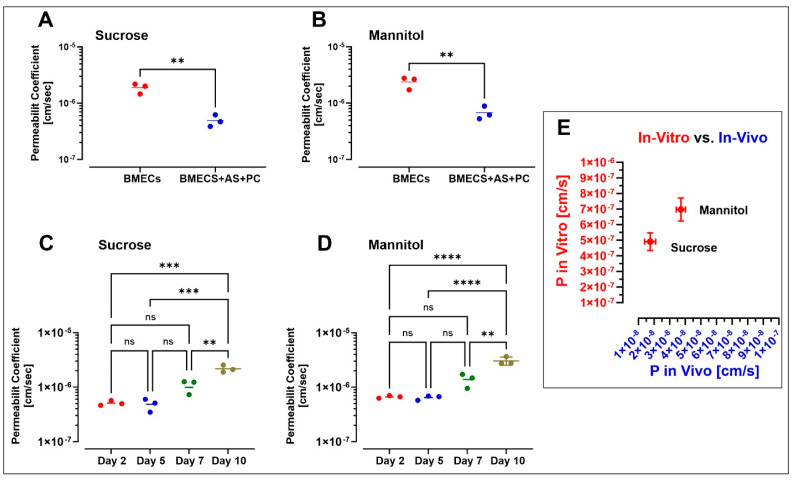
The permeability coefficient of sucrose (**A**) and mannitol (**B**) in BBB on a chip with iBMECs alone and iBMECs with primary human astrocytes and pericytes cultured under continuous flow (0.15 dyne/cm^2^) for 2 days. Student’s *t*-test (*p* < 0.01); *n* = 3 biological replicates. Long-term stability of the barrier function by a comparison of the permeability coefficients of sucrose (**C**) and mannitol (**D**) on different days under continuous flow (0.15 dyne/cm^2^). ** *p* < 0.01, *** *p* < 0.001, and **** *p* < 0.0001, assessed by one-way ANOVA, followed by Tukey’s multiple comparisons test (*n* = 3 biological replicates). (**E**) Comparison of sucrose and mannitol permeability assessed in our BBB microfluidic model with previously reported in vivo data in mice (*n* = 3 biological replicates).

We assessed the passage of [^13^C_12_] sucrose and [^13^C_6_] mannitol, known as one of the ideal paracellular permeability markers [23,32], across iBMEC cell monolayers cultured in single and triculture models under a continuous flow condition (0.15 dyne/cm^2^) to investigate the difference in barrier properties. Results show that the permeability of [^13^C_12_] sucrose and [^13^C_6_] mannitol across the single culture of the iBMEC monolayer was 1.883 × 10^−6^ ± 3.763 × 10^−7^ and 2.380 × 10^−6^ ± 5.738 × 10^−7^ cm/s (*n* = 3 biological replicates), respectively, after 2 days in the culture, while the permeability of sucrose and mannitol in the triculture was 4.923 × 10^−7^ ± 1.187 × 10^−7^ and 6.760 × 10^−7^ ± 1.071 × 10^−7^ cm/s (*n* = 3 biological replicates), respectively (see Figure 3A,B). Compared with iBMECs cultured alone, a triculture with human pericytes and astrocytes significantly decreased the blood-to-brain paracellular leakage of sucrose and mannitol (*p* < 0.01 unpaired, two-tailed *t*-test). Accordingly, our results strongly suggest that a co-culture with primary human astrocytes and pericytes can enhance the barrier function of the BBB on a chip. Furthermore, permeability measurements were performed at 2, 5, 7, and 10 days post-setup (see Figure 3C,D) to assess the longitudinal viability of the microfluidic BBB system. Our data show stable BBB integrity up to 7 days, at which point, the permeability coefficients of sucrose and mannitol started increasing. For instance, the permeability coefficient of sucrose was 5.093 × 10^−7^ ± 5.395 × 10^−8^ and 5.317 × 10^−7^ ± 1.628 × 10^−7^ cm/s on day 2 and day 5 (*n* = 3 biological replicates), respectively, whereas it was 1.166 × 10^−6^ ± 3.277 × 10^−7^ and 2.180 × 10^−6^ ± 3.306 × 10^−7^ cm/s on day 7 and day 10 (*n* = 3 biological replicates), respectively. The permeability on days 2 and 5 was significantly different from that measured on day 10 (*p* < 0.001 one-way ANOVA, followed by Tukey’s multiple comparisons test). A similar trend was observed when measuring mannitol permeability (*n* = 3), which increased from 6.640 × 10^−7^ ± 3.651 × 10^−8^ and 6.430 × 10^−7^ ± 3.557 × 10^−8^ on day 2 and day 5 to 3.053 × 10^−6^ ± 5.258 × 10^−7^ on day 10 (*p* < 0.0001 one-way ANOVA, followed by Tukey’s multiple comparisons test).

Previously, we reported the permeability coefficient of these markers in mouse models through IV bolus injection [23]. We observed a correlation between our current in vitro and the ones we obtained in vivo. The permeability coefficient of sucrose and mannitol in-vivo (*n* = 3) was 1.75 ± 0.355 × 10^−8^ and 3.71 ± 0.296 × 10^−8^ cm/s, respectively. Sucrose and mannitol (permeability coefficient values) in microfluidic chips were only 1 log magnitude higher than those assessed in vivo (Figure 3E).

### 3.3. Effect of Microfluidic Shear Stress on BBB Integrity and Barrier Function

**Figure 4:**
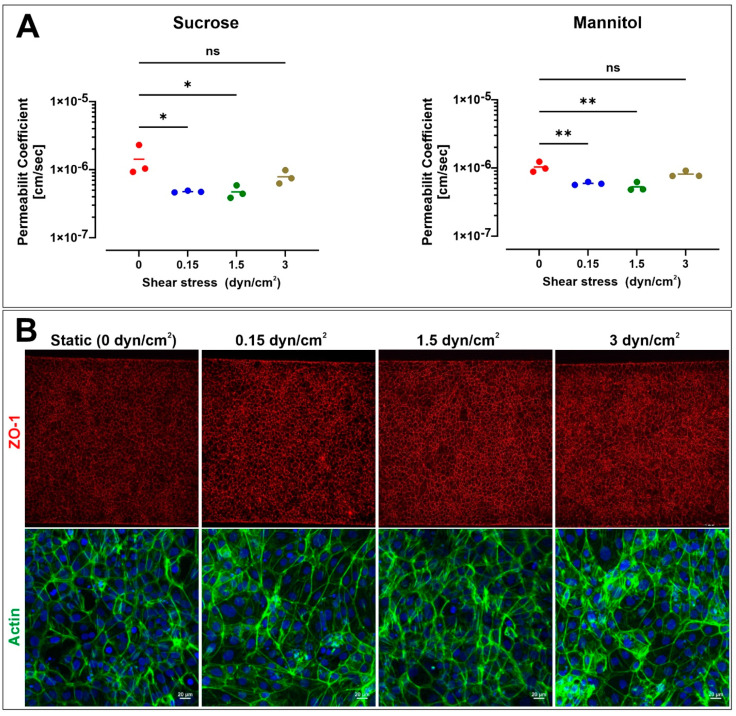
Effect of shear stress on the permeability coefficient of sucrose and mannitol in a microfluidic chip. (**A**) Note the effect of shear stress on the permeability of both paracellular markers. (**B**) Representative immunofluorescence images of iBMEC monolayers fixed and stained after 48 h at 0, 0.15, 1.5, and 3 dyne/cm^2^. ZO-1 (red) and F-actin phalloidin (green). *n* = 3 biological replicates. * *p* < 0.05 and ** *p* < 0.001 assessed by one-way ANOVA, followed by Tukey’s multiple comparisons test.

We studied the effect of shear stress on BBB permeability using the microfluidic BBB chip. iBMECs and primary human pericytes and astrocytes were seeded on vascular and brain channels and allowed to attach under static (no flow) conditions for 1 day. After that, platforms were either maintained under static conditions with daily media changes or exposed to flow using a peristaltic pump at a rate of 120, 1200, or 2400 µL/h (equivalent to 0.15, 1.5, and 3 dyn/cm^2^) for 48 h. Based on permeability measurements, the integrity and tightness of the BBB slightly increased in the presence of flow compared to static conditions. For instance, the permeability coefficient of sucrose (*n* = 3 biological replicates; see Figure 4A, left panel) was 1.521 × 10^−6^ ± 4.707 × 10^−7^ cm/s in static conditions without any perfusion on day 3. In contrast, the permeability values decreased to 5.093 × 10^−7^ ± 1.595 × 10^−8^, 5.05 × 10^−7^ ± 1.125 × 10^−7^, and 8.4 × 10^−7^ ± 1.931 × 10^−7^ cm/s in response to the exposure to shear stress of 0.15, 1.5, and 3 dyn/cm^2^, respectively. Similar statistically significant results were obtained for mannitol permeability (*n* = 3 biological replicates), ranging from 1.105 × 10^−6^ ± 1.939 × 10^−7^ cm/s in static conditions (see Figure 4A right panel) to 6.337 × 10^−7^ ± 1.898 × 10^−8^, 5.67 × 10^−7^ ± 4.952 × 10^−8^, and 8.713 × 10^−7^ ± 5.184 × 10^−8^ cm/s in response to shear stress of 0.15, 1.5, and 3 dyn/cm^2^, respectively. These results are in agreement with previous work highlighting the role of shear stress in BBB endothelial physiology [33] and support the notion that exposure to shear stress enhances the barrier tightness, resulting in lower paracellular permeability. Figure 4B shows the fluorescence image of the tight-junction protein ZO-1 and f-actin phalloidin on day 3 in static and dynamic conditions. Under static conditions, ZO-1 was localized to cell–cell junctions and showed a polygonal network, consistent with previous reports on the iBMEC monolayer [34]. There were no clear differences between ZO-1 stains under static and flow conditions, suggesting that tight-junction networks are already formed under static conditions. Additional quantitative experiments will be necessary to better understand the effect of shear stress on the tight junctions and the cytoskeleton.

Furthermore, F-actin expression indicated substantial intercellular localization in iBMEC-lined channels. The patterns exhibit the lack of cellular elongation in cells exposed to fluidic shear. This property has been reported previously to be a unique feature of iBMECs [18,34]. F-actin continued to be randomly oriented and did not align parallel to flow in all the groups.

### 3.4. BBB-on-a-Chip Responds to P-gp Inhibition

**Figure 5:**
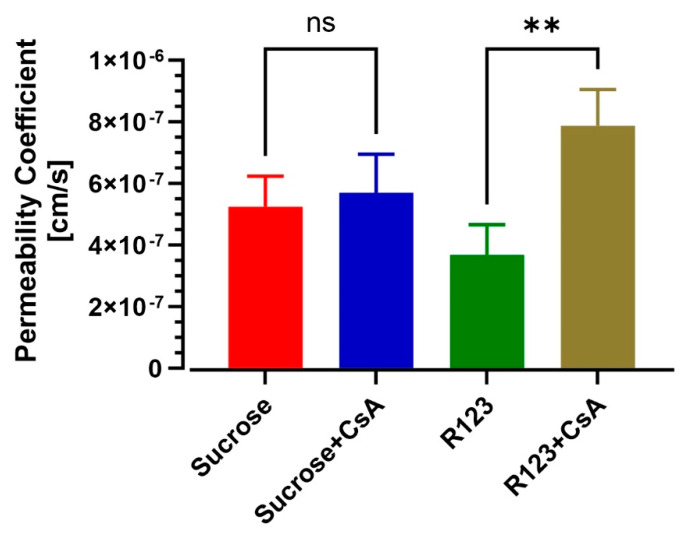
Assessment of efflux transporter functionality of iBMECs in BBB on a chip with and without cyclosporine A treatment. Note the permeability coefficient of rhodamine 123 (substrate of P-gp transporter) and sucrose in the presence or absence of the P-gp inhibitor (*n* = 3 biological replicates). ** *p* < 0.01, assessed by one-way ANOVA, followed by Tukey’s multiple comparisons test.

We investigated the ability of the model to replicate the efflux transporter activity and act as a metabolic barrier. P-gp is expressed on the endothelium and acts as an efflux pump to protect the brain from unwanted molecules. To study P-gp efflux inhibition, 5 µM cyclosporine A was supplemented in channels. Then the permeability of sucrose and rhodamine 123 (substrate of P-gp transporter) was evaluated in the presence or absence of the inhibitor. As shown in Figure 5**,** treatment with P-gp inhibitor cyclosporine A (CsA) increased R123 permeability from 3.677 × 10^−7^ ± 5.669 × 10^−8^ to 7.876 × 10^−7^ ± 6.791 × 10^−8^ cm/s (*n* = 3) (*p* < 0.01 unpaired, two-tailed *t*-test), while the permeability of sucrose was not altered by CsA (*p* > 0.50 unpaired, two-tailed *t*-test).

### 3.5. Hyperosmolar BBB Opening

**Figure 6:**
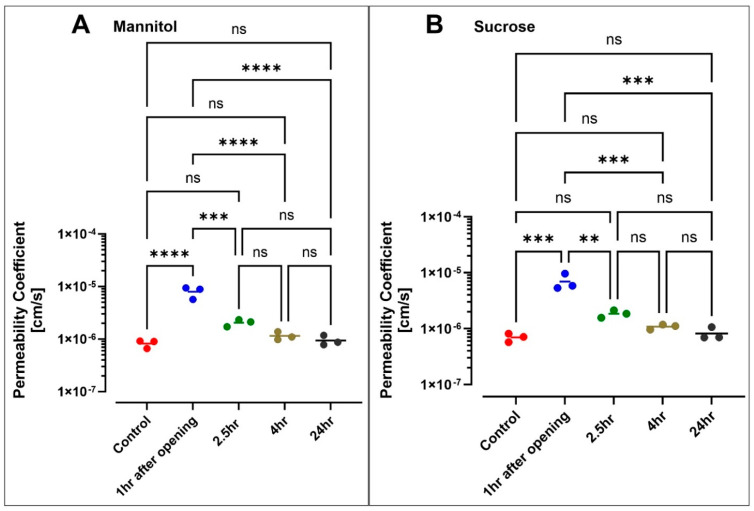
Evaluation of the barrier integrity of the BBB chip after exposure to hyperosmolar mannitol for 10 min. Monitoring the permeability of sucrose (**A**) and mannitol (**B**) over a 24 h window after exposure to hyperosmolar mannitol revealed a significant increase in their respective permeability values at 1 h post-opening. Sucrose and mannitol permeability then returned progressively to normal over the 24 h period (*n* = 3 biological replicates). ** *p* < 0.01, *** *p* < 0.001, and **** *p* < 0.0001 assessed by one-way ANOVA, followed by Tukey’s multiple comparisons tests.

We infused a hyperosmolar mannitol solution into the vascular channel for 10 min. We then immediately perfused the lumen with [^13^C_12_] sucrose and [^13^C_6_] mannitol to assess the impact of the hyperosmolar challenge on BBB integrity as a function of permeability. Figure 6A,B shows that the transport of markers increased to the brain channel after exposure to hyperosmolar mannitol. The permeability coefficient of sucrose increased from 6.978 × 10^−7^ ± 1.22 × 10^−7^ cm/s pre-opening to 6.910 × 10^−6^ ± 2.014 × 10^−6^ cm/s 1 h following the hyperosmolar challenge. A similar trend was observed for mannitol permeability (increasing from 8.253 × 10^−7^ ± 1.436 × 10^−7^ cm/s pre-opening to 7.983 × 10^−6^ ± 2.398 × 10^−6^ cm/s 1 h post-opening). A longitudinal assessment of paracellular permeabilities showed that the BBB integrity started recovering at 2.5 h after the hyperosmolar challenge (P_SUC_ = 1.840 × 10^−6^ ± 2.800 × 10^−7^ cm/s; P_MAN_ = 2.057 × 10^−6^ ± 3.099 × 10^−7^ cm/s) and was fully restored at 24 h (P_SUC_ = 8.163 × 10^−7^ ± 2.11 × 10^−7^ cm/s; P_MAN_ = 9.437 × 10^−7^ ± 2.097 × 10^−7^ cm/s).

## 4. Discussion

Small-molecular-weight markers, such as sucrose, mannitol, and sodium fluorescein, have naturally low BBB permeability; therefore, these markers have been used widely to assess the integrity of the BBB. Recently published studies have emphasized the alternative use of dextran molecules of relatively large molecular weights (3 to 70 kDa) to assess the integrity and tightness of the BBB [4,18,19]. However, large-molecular-weight markers may not accurately assess the integrity and tightness of the BBB in permeability studies involving small, drug-like molecules. In this study, we used a recently developed method that quantifies the stable isotopes of sucrose and mannitol with high sensitivity and accuracy for measuring BBB integrity [23]. Sucrose and mannitol may be considered the most widely accepted markers for the precise measurement of paracellular BBB permeability in vivo due to their unique characteristics, such as being uncharged and remaining largely unbound from proteins. Therefore, we characterized the structure and barrier function of a novel BBB microfluidic chip established using human iBMECs with or without the presence of additional NVU components, including astrocytes and pericytes. We noticed that the model maintained the barrier function for at least 7 additional days, enabling time-course studies of biologically and clinically relevant variations. [^13^C_12_] sucrose and [^13^C_6_] mannitol permeability values in a microfluidic chip were 4.923 × 10^−7^ ± 1.187 × 10^−7^ and 6.760 × 10^−7^ ± 1.071 × 10^−7^ cm/s (see Figure 3A,B), respectively, which were quite close to their corresponding values recorded in vivo [23].

The barrier function of some recently published iPSC-based BBB microfluidic models was reported to be comparable to in vivo values [17,18,21,32]. These data were obtained by correlating the permeability coefficients of dextran in these models against rat pial post-capillaries using imaging studies [22]. The permeability coefficient values of dextran (4–70 kDa) in rat pial post-capillaries were reported in the range of 1 to 9 × 10^−7^ cm/s. For instance, the permeability coefficient of 10 kDa dextran was shown to be 3.1 × 10^−7^ cm/s in rat cerebral microvessels. A drawback of such imaging techniques is the lack of quantitative uptake measurement and vessel damage resulting from the grounding of the skull. As a result, these techniques could lead to the overestimation of dextran permeability levels. Studies, for example, by Mehvar et al., have demonstrated that even with 4 kDa dextran, there is no fluorescein-labeled dextran concentration in brain tissue [35]. Similarly, Linville et al. also developed an advanced BBB microvessel, demonstrating that 10 kDa dextran does not pass across the BBB and that the permeability is below the detection limit [21].

We also found that a co-culture with human pericytes and astrocytes significantly decreases the permeability values of sucrose and mannitol compared to iBMECs alone. This finding exemplifies the notion that our multi-culture model provides a realistic cellular environment for the iBMECs to form a viable and effective BBB in vitro. Vatine et al. and Park et al. found similar results when comparing the permeability of 3 kDa dextran in a monoculture and a triculture in microfluidic chips [18,19]. Moreover, Park et al. found that the mRNA levels of ZO-1, VE-cadherin, and MRP1 are remarkably higher in iBMECs when co-cultured with brain astrocytes and pericytes on a chip [19].

The morphology of iBMECs is not changed in the presence of laminar flow, which indicates that these cells do not elongate and align as a response to shear stress. Furthermore, immunofluorescence images of tight junctions show similar patterning and expression levels, implying that laminar flow is unnecessary for iBMECs to establish a tight barrier. DeStefano et al. confirmed that shear stress does not induce any changes in the expression of several BBB markers at the protein or gene level [34], whereas Vatine et al. showed that ZO-1 gene expression is increased in all laminar flow conditions, including low shear stress (0.01 dyn/cm^2^) [18]. We confirmed that shear stress decreases the permeability values of mannitol and sucrose. The effect is not drastic but still statistically significant when compared to static conditions. These findings are in agreement with DeStefano et al.’s conclusions, which suggests that iBMECs can establish a barrier function under static conditions, but also are in agreement with previous studies from our group supporting a positive barrier modulatory role of shear stress [33]. However, additional studies will be required to determine the environmental contribution of astrocytes and/or pericytes to the iBMECs expression and distribution of the TJs and further confirm the role of shear stress in the process since results from prior studies using iBMECs have yielded controversial results [18,34,36]. In addition, whether and to what degree the observed phenomenon applies to primary BMEC under similar multi-culture conditions will need to be determined. Human brain microvascular endothelial cells (HBMECs) in the presence of shear stress demonstrate upregulation of tight and adherens junction proteins and the development of a more stringent (high TEER values) barrier compared to static conditions [33].

iBMECs in a monoculture exhibit many human BBB properties, including well-organized tight junctions, polarized efflux transporter activity, and high TEER values (1000–3000 Ω·cm^2^). However, a significant limitation of these cells hindering their usability for targeted CNS drug delivery and time-course studies is the short-lived BBB viability, which is maintained for about 2 days [29,37]. Our study clearly showed that using a dynamic multi-culture setting encompassing astrocytes, pericytes, and intraluminal flow can further extend the barrier viability up to 5–7 days.

Systemic intravenous injection of the hyperosmolar agent mannitol is used clinically to reduce cerebral edema in acute conditions [38]. In contrast, at a high concentration (25% *w*/*v*), bolus intra-arterial injection of mannitol results in endothelial cell shrinkage, leading to a transient opening of the BBB [39,40,41]. Hyperosmolar BBB opening has been used in clinical and translational settings to facilitate the delivery of chemotherapeutics, stem cells, and viral vectors into the brain [40,42,43]. Herein, we tested the response of our BBB-on-a-chip model to similar osmotic challenges. Following a 10 min infusion of a bolus 25% mannitol, we recorded an eightfold increase in ^13^[C_12_] sucrose permeability, which closely mimics previous results obtained by our group in vivo using a mouse model undergoing transient BBB opening by hyperosmolar mannitol [44]. Four hours post-hyperosmolar opening, the integrity of the BBB was restored, as indicated by sucrose and mannitol permeability measurement (see Figure 6). Our data are in line with previous results from Linville et al. reporting that hyperosmolar mannitol causes transient focal leaks that result in a significant permeability increase of Lucifer yellow and 10 kDa dextran [21,45]. They also confirmed the recovery of barrier integrity 3 h post-infusion [45]. Higher penetration of both 10 kDa dextran and the cetuximab antibody into the brain channel was also reported by Park et al. in response to the osmotic opening of the BBB in a microfluidic chip. Within 4 h post-opening, the permeability of dextran and the antibody declined to the normal (pre-opening) range, indicating a recovery of the barrier integrity [19].

ATP-binding cassette efflux transporters, including P-glycoprotein (P-gp), limit the penetration of many lipophilic compounds into the brain parenchyma by actively transporting them out of the brain [45]. Hence, modulation of efflux transporters plays a crucial role in controlling CNS drug delivery. Accordingly, numerous reports have demonstrated that iBMECs possess active efflux activity as determined by substrate inhibition assays [19,21,29,37]. Here, we sought to characterize this efflux activity in a 3D microfluidic chip. The permeability of rhodamine 123 (a P-gp substrate) was evaluated in the presence or absence of cyclosporin A (a P-gp inhibitor) in iBMECs. Cyclosporine A is a P-gp inhibitor used in human studies to assess P-gp activities [46,47]. We showed that the permeability of R123 increases by about twofold, while the barrier’s paracellular permeability to sucrose remains unaltered. These results indicate that our microfluidic model can reproduce a crucially important feature of the BBB affecting the passage of drugs into the brain, thus playing a significant role in determining drug permeability tests’ predictive value and reliability. Additional testing will be required to further assess the viability of other major efflux transporters, including BCRP and MRP1, and more permeability testing using clinically relevant CNS drugs with known permeability coefficients will be required to better assess the predictive value of our model.

## 5. Conclusions and Future Studies

This study validated the barrier function of a human iPSC-derived blood-brain barrier microfluidic chip by using small-molecular-weight markers. Sucrose and mannitol are known as ideal standard markers for the measurement of BBB integrity in in vivo studies. We showed low permeability coefficient values for [^13^C_12_] sucrose and [^13^C_6_] mannitol in the microfluidic model. The BBB microfluidic model displays critical tight-junction proteins, an efflux pump, and transporters. Moreover, the physiological barriers were maintained until day 7, which could be used for any chronic disease studies. We also demonstrated successful modulation of both transcellular and paracellular permeability using the P-gp inhibitor and hyperosmolar agent mannitol. Our results suggest that the multi-culture microfluidic model can effectively reproduce a quasi-physiological microenvironment, allowing for developing a highly predictive and translationally relevant BBB model. This novel platform can be a valuable tool for screening putative CNS-targeting drugs and assess the feasibility and effectiveness of novel delivery methods across the BBB.

## Data Availability

Not Applicable.

## Supplementary Materials

The following are available online at https://www.mdpi.com/article/10.3390/pharmaceutics13091474/s1: Figure S1: Top view of immunofluorescence micrographs of the channel with low magnification. (**A**) ZO-1, (**C**) GLUT-1, and (**E**) P-gp. Side view of the channel highlighting ZO-1 (**B**) and GLUT-1 (**D**), Table S1: Antibodies used for immunofluorescence studies in BBB on a chip.

## Abbreviations

3D	three dimensional
BBB	blood-brain barrier
iBMECs	human brain microvascular endothelial cells derived from iPSCs
BMECs	brain microvascular endothelial cells
CNS	central nervous system
CsA	cyclosporine A
DAPI	4′,6-diamidino-2-phenylindole
ECM	extracellular matrix
GFAP	glial fibrillary acidic protein
GLUT-1	glucose transporter
iPSC	induced pluripotent stem cells
LC-MS	liquid chromatography and mass spectrometry
MRP	multidrug-resistance-associated protein
NVU	neurovascular unit
PDMS	polydimethylsiloxane
P-gp	P-glycoprotein
R123	rhodamine 123
TEER	trans-endothelial electrical resistance
TJs	tight junctions
UPLC	ultra-performance liquid chromatography
ZO-1	zona occludens-1
α-SMA	alpha-smooth muscle actin
