# Supplementary material for: A Quasi-Physiological Microfluidic Blood-Brain Barrier Model for Brain Permeability Studies
Source: Pharmaceutics. 2021 Sep 15;13(9):1474. doi: 10.3390/pharmaceutics13091474 (PMC8468926; doi:10.3390/pharmaceutics13091474)
Supplement: Supplementary file 1 [file pharmaceutics-13-01474-s001.zip › pharmaceutics-1373702-supplementary.pdf]

pharmaceutics

MDPI

Supplementary Materials: A Quasi-Physiological Microfluidic Blood-Brain Barrier Model for Brain Permeability Studies

Behnam Noorani, Aditya Bhalerao , Snehal Raut, Ehsan Nozohouri, Ulrich Bickel and Luca Cucullo

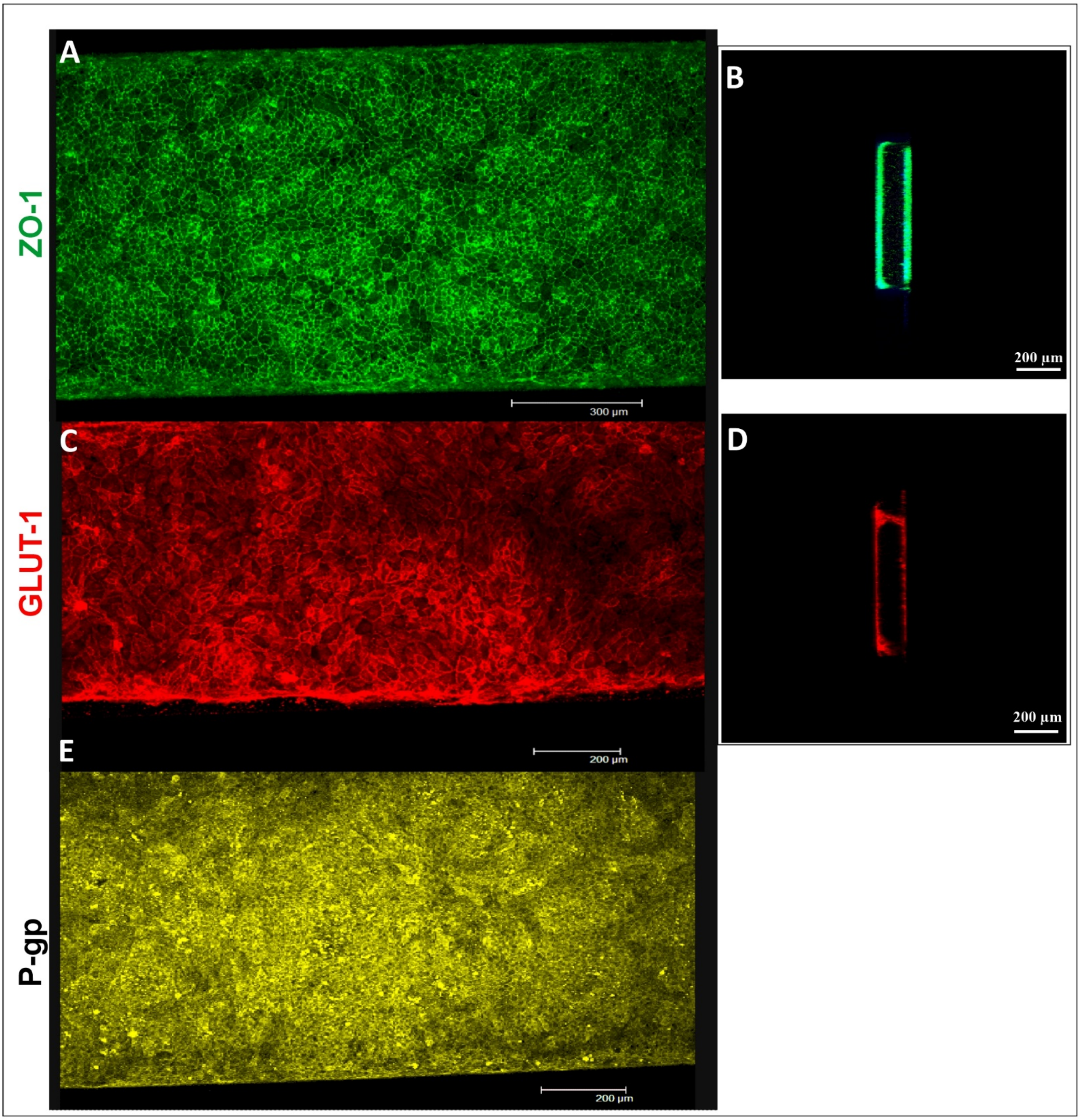

Figure S1. Top view of immunofluorescence micrographs of the channel with low magnification. (**A**) ZO-1; (**C**) GLUT-1; (**E**) P-gp. Side view of the channel highlighting ZO-1 (**B**) and GLUT-1 (**D**).

Table S1. Antibodies used for immunofluorescence studies in BBB-on-a-chip.

Targeted Antigen	Antibody Description	Vendor	Dilution
ZO-1	Polyclonal rabbit	Invitrogen	1:100
Claudin-5	Monoclonal Mouse	Invitrogen	1:100
GLUT-1	Monoclonal Mouse	Thermofisher	1:100
p-glycoprotein	Monoclonal Mouse	Thermofisher	1:50
GFAP	Monoclonal Mouse	Cell signaling	1:200
α-Smooth Muscle Actin	Polyclonal rabbit (Alexa Fluor® 555 Conjugate)	Cell signaling	1:200
F-actin	Alexa Fluor™ 568 Phalloidin	Thermofisher	5:200
